# Expression of LRIG proteins as possible prognostic factors in primary vaginal carcinoma

**DOI:** 10.1371/journal.pone.0183816

**Published:** 2017-08-25

**Authors:** Cecilia Ranhem, Gabriella Lillsunde Larsson, Håkan Hedman, David Lindquist, Mats G. Karlsson, Ann-Cathrin Hellström, Ellinor Östensson, Bengt Sorbe, Kristina Hellman, Sonia Andersson

**Affiliations:** 1 Department of Women’s and Children’s Health, Karolinska Institutet, Stockholm, Sweden; 2 Region Västmanland – Uppsala University, Centre for Clinical Research, Hospital of Västmanland Västerås, Västerås, Sweden; 3 School of Health and Medical Sciences, Örebro University, Örebro, Sweden; 4 Department of Laboratory Medicine, Örebro University Hospital, Örebro, Sweden; 5 Department of Radiation Sciences, Umeå University, Umeå, Sweden; 6 Department of Laboratory Medicine, Faculty of Medicine and Health, Örebro University, Örebro, Sweden; 7 Department of Medical Epidemiology and Biostatistics, Karolinska Institutet, Stockholm, Sweden; 8 Department of Oncology, Örebro University Hospital, Örebro, Sweden; 9 Department of Oncology-Pathology, Karolinska Institutet, Stockholm, Sweden; Fondazione IRCCS Istituto Nazionale dei Tumori, ITALY

## Abstract

**Background:**

Primary vaginal carcinoma (PVC) is a rare malignancy. Established prognostic factors include tumour stage and age at diagnosis. The leucine-rich repeats and immunoglobuline-like domains (LRIG)-1 protein functions as a tumour suppressor, but less is known about the functions of LRIG2 and LRIG3. The present study aimed to evaluate the expression of LRIG proteins and analyse their possible associations with clinical characteristics and survival in a cohort of PVC patients.

**Methods:**

We used immunohistochemistry to investigate LRIG1, LRIG2, and LRIG3 expression in tumour samples from a consecutive cohort of 70 PVC patients. The association between LRIG protein expression and clinical characteristics and cancer-specific survival was investigated using univariate and multivariate analyses.

**Results:**

The majority of PVC patients (72%) had >50% LRIG1- and LRIG2-positive cells, and no or low LRIG3-positive cells. HPV status was significantly correlated with LRIG1 expression (p = 0.0047). Having high LRIG1 expression was significantly correlated with superior cancer-specific survival in univariate and multivariate analyses. LRIG2 and LRIG3 expression did not significantly correlate with clinical characteristics or survival.

**Conclusion:**

LRIG1 expression might be of interest as a prognostic marker in PVC patients, whereas the role of LRIG2 and LRIG3 expression remains to be clarified.

## Introduction

Primary vaginal carcinoma (PVC) is a rare malignancy of the female genital tract. It has a poor prognosis and most commonly affects postmenopausal women over 60 years of age [1–3]. Survival rates depend on patient age and clinical stage of the disease, but overall survival is less than 50% [1,2]. The most common histological subtype of PVC is squamous cell carcinoma; adenocarcinomas are also seen, but other histological subtypes are very rare [3]. Studies on PVC are scarce and small due to the rarity of the disease, and increased knowledge of biological and prognostic factors is required in order to improve clinical strategies for these patients.

Studies on the treatment of PVC are also rare and have shown inconclusive results. Given the relative frequency of cervical cancer, as well as its anatomical, histological, and biological similarities with PVC, treatment strategies for PVC tend to be extrapolated from those for cervical cancer [4]. Therefore, most PVC patients are treated with radiotherapy [5] or definitive chemoradiotherapy [6,7], which sometimes results in substantial toxic side effects.

Similarly to other squamous cell carcinomas of the genital tract, the majority of PVC cases are related to human papillomavirus (HPV) infection [3,8–13]. Although it has been suggested that HPV could be a useful prognostic marker for PVC [8,12], another study was unable to confirm this [14], and yet another study confirmed it only in women with advanced stages of disease [15]. Other prognostic factors that have repeatedly been shown to be significant in PVC include clinical variables such as age, tumour size [16,17], and clinical stage [2,8]. Proposed prognostic biomarkers have included p16, Ki67, p53, and laminin-5 expression [14,16,17].

The expression of the human leucine-rich repeats and immunoglobulin-like domains (LRIG) proteins LRIG1, LRIG2, and LRIG3 has emerged as a new potential prognostic biomarker in different types of human cancer, including cervical cancer [18]. LRIG1, LRIG2, and LRIG3 encode transmembrane proteins involved in the regulation of growth factor signalling and cell proliferation. Accordingly, the expression of LRIG proteins is commonly dysregulated in human cancer. LRIG1 has been proposed to function as a tumour suppressor through negative regulation of oncogenic receptor tyrosine kinases, such as members of the ERBB family, MET and RET receptors, and PDGFRA [19]. LRIG1 expression is of prognostic significance and is associated with good prognosis in various human cancers, including cervical cancer [20,21], non-small-cell lung cancer [22,23], breast cancer [24], and others [18]. In cervical cancer, the variation in the number of LRIG1 gene copies and promoter methylation patterns are associated with patient survival [25]. Although we know that LRIG3 interacts with, and possibly opposes the function of, LRIG1 [26], we know little else about it, or its counterpart LRIG2. In general, high expression of LRIG1 and LRIG3 in tumours is associated with improved survival in cancer patients, whereas high expression of LRIG2 is associated with poor survival even in early-stage cervical cancer patients [27]. Previous studies on cervical cancer have implied that each LRIG protein may be of different prognostic value, depending on histology and stage of disease [20,21,27].

The LRIG proteins have not previously been studied in PVC. The present study aimed to evaluate the expression of LRIG proteins and analyse their possible associations with clinical characteristics and survival in a cohort of PVC patients.

## Materials and methods

The present analysis used archived PVC samples collected from a consecutive cohort of 81 patients with PVC treated at Örebro University Hospital, and at the central hospitals in Eskilstuna, Västerås, and Karlstad between 1975 and 2002. In a previous study by Larsson *et al*, [12] data on age, tumour site, FIGO stage, tumour localisation, histology (based on World Health Organisation criteria and included basaloid squamous cell carcinoma, non-keratinizing squamous cell carcinoma, keratinizing squamous cell carcinoma, verrucous squamous cell carcinoma, adenocarcinoma, sarcoma, and melanoma), tumour grade, and treatment for each patient were recovered from hospital records and were used in this analysis. We also used follow-up data from Larsson *et al* [12]. All patients were retrospectively followed up from the time of diagnosis and median follow-up time for patients who were alive at the end of the study was 121 months (range 44–290 months). Complete remission was defined as the disappearance of all clinical evidence of disease after primary treatment. Tumour recurrence was defined as the detection of cancer after a period of at least 6 months of initial complete remission. Finally, we used information on HPV status reported by Larsson *et al* [12]. In that study, all 81 samples were subjected to HPV testing and genotyping. Among the 81 PVC cases, 37 were HPV-positive, 34 were HPV-negative and 10 had insufficient material for HPV detection (Table 1). Of the 37 HPV-positive cases, 26 (70%) were HPV16-positive, whereas the remaining 11 were positive for other high-risk HPV genotypes.

**Table 1:** Patient and tumour characteristics (n = 81).

	**Mean (range)**
**Age (years)**	69 (37–90)
**Tumour size (cm)**	2.5 (1.0–4.0)
	
**FIGO stage**	**N (%)**
**I**	25 (31)
**II**	34 (42)
**III**	9 (11)
**IV**	13 (16)
	
**Localisation**	
Upper vagina	23 (28)
Middle vagina	14 (17)
Middle-lower	2 (3)
Lower vagina	26 (32)
Entire length of vagina	16 (20)
	
**Histology**	
Squamous cell carcinoma	72 (89)
Basaloid	28
Non-keratinizing	24
Keratinizing	9
Verrucous	2
Adenocarcinoma	7 (8.5)
Other	2 (2.5)
	
**Tumour grade**	
1	13 (16)
2	30 (37)
3	27 (33)
Unknown	11 (14)
	
**HPV status**	**(n = 71)**
Negative	34 (48)
Positive	37 (52)
HPV 16	26
Other HPV types	11
	
**Treatment**	
EBRT*	10 (12.5)
EBRT+BT	48 (59)
BT**	13 (16)
Other	10 (12.5)

*EBRT = external beam radiotherapy,

**BT = vaginal brachytherapy

The present study was approved by the regional Ethical Committee in Uppsala, Sweden (EPN, Dnr 2008/294). Patients were orally informed about the clinical research database, and after 2003 they were also informed about tissue biobanking according to the Swedish biobank act 2002:297. No specific informed consent was requested by the Ethical Committee.

### Immunohistochemistry for LRIG protein expression

Seventy of the 81 PVC patients had sufficient material for immunohistochemical analyses. Immunohistochemical staining for LRIG1, LRIG2, and LRIG3 was carried out on formalin-fixed paraffin-embedded sections of 3.5–4 μm. Tissue sections were incubated for 60 minutes at 60°C. Immunohistochemical staining was performed in an automated Ventana Benchmark XT apparatus (Ventana Medical Systems, Tucson, AZ, USA) using rabbit primary antibodies for LRIG1, LRIG2, and LRIG3 [28–31]. Epitope retrieval for LRIG1 and LRIG2 was done with Cell conditioning 1 (Product nr. 950–124, Ventana Medical Systems) for 60 minutes at 95°C. For LRIG 3, Protease 1 (Product nr. 760–2018, Ventana Medical Systems) was used for 12 minutes at 37°C. Automated immunohistochemical staining was performed using the ultraView Universal DAB Detection Kit (Product nr. 760–500, Ventana Medical Systems). The slides were deparaffinised and blocked for peroxidase using ultraView Universal DAB Inhibitor containing 3% hydrogen peroxide solution. Primary antibodies (LRIG1; 1:100, LRIG 2; 1:200, and LRIG 3; 1:50) were incubated for 32 minutes at 37°C. UltraView Universal HRP Multimer, ultraView Universal DAB Chromogen, and ultraView Universal Copper were used according to the manufacturer’s instructions. The slides were counterstained with hematoxylin (Product nr. 760–2021, Ventana Medical Systems) for 4 minutes followed by bluing with Bluing Reagent (Product nr. 760–2037, Ventana Medical Systems) for 4 minutes, both at 37°C.

A senior pathologist (MK), blinded to all clinical data, evaluated the immunostaining. The percentage of positive cells was based on a 4-point semi-quantitative scale: 0 = 0% positive cells, 1 = 1–25% positive cells, 2 = 25–50% positive cells, and 3 = >50% positive cells. Cells were considered positive regardless of whether the staining was cytoplasmic or nuclear (see S1 Table for description of LRIG protein expression and HPV status for all patients).

### Statistical analysis

Associations between ordinal variables were tested using a Pearson chi-square or Fisher’s exact test. Patient and tumour characteristics, HPV status, and LRIG1, LRIG2, and LRIG3 expression were illustrated in a Kaplan-Meier graph to reflect possible prognostic factors for cancer-specific survival, and a log-rank test was used to compare different groups. Age, tumour size, FIGO stage, and HPV status were significant in the univariate analysis, and thus were included in a Cox regression multivariate analysis. All significance testing was carried out at the 0.05 level using SPSS 19 software.

## Results

### LRIG protein expression, patient and tumour characteristics, and HPV status

**Fig 1:**
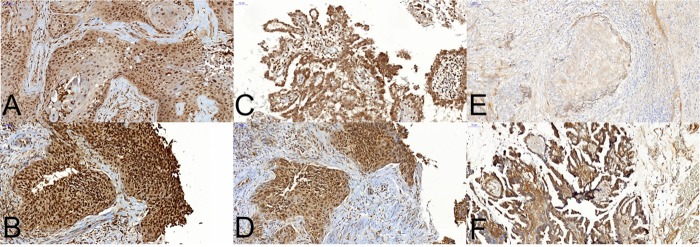
Examples of different staining intensities for LRIG1, LRIG2, and LRIG3 in primary vaginal carcinoma.

A majority of the 70 tumours showed LRIG1 and LRIG2 expression in >50% of cells and demonstrated no or low LRIG3 expression (Table 2). Fig 1 shows examples of the evaluation of staining intensity.

**Table 2:** Immunohistochemical staining of LRIG1, LRIG2, and LRIG3 (n = 70).

Immunohistochemical score*	N (%)
**LRIG1**	
0	0 (0)
1	0 (0)
2	12 (17)
3	58 (83)
**LRIG2**	
0	0 (0)
1	0 (0)
2	12 (17)
3	58 (83)
**LRIG3**	
0	37 (53)
1	29 (41)
2	4 (6)
3	0 (0)

*Protein score: 0 = 0%, 1 = 1–25%, 2 = 25–50%, 3 = >50% of positive cells.

High LRIG1 expression was significantly correlated with HPV positivity (Pearson chi-square; p = 0.0047). No other statistically significant correlations were found between LRIG expression, tumour size, FIGO stage, localisation, histology, tumour grade or HPV status. Among non-keratinizing tumours, 66.7% were HPV-positive, compared to only 22.8% of keratinizing tumours (Pearson chi-square: p = 0.022).

### Primary cure rate and cancer-specific survival rates

**Fig 2:**
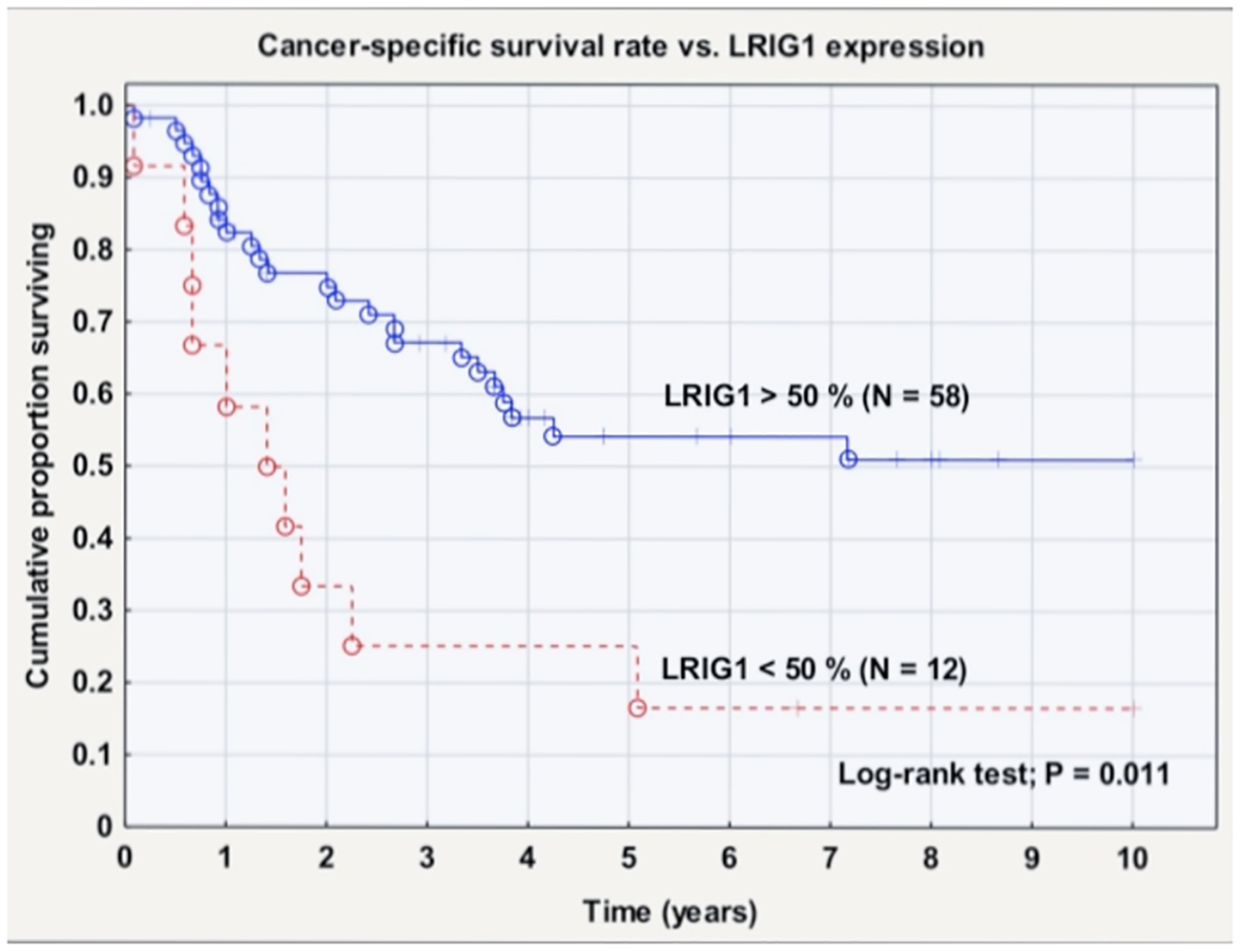
Cancer-specific survival rate versus LRIG1 expression in immunohistochemical staining (score 3 vs. score 0–2). Log-rank test showed a statistically significant difference (p = 0.011).

High LRIG1 expression (>50% vs <50% positive cells) correlated with a higher primary cure rate (Pearson chi-square, p = 0.0004). High LRIG1 expression (>50% vs <50% positive cells) was also associated with a significantly better survival rate (log-rank test: p = 0.011), and this difference was most pronounced in HPV-negative PVC (log-rank test: p = 0.027). There was no statistically significant correlation between LRIG2 or LRIG3 expression and patient survival (Table 3, Fig 2).

**Table 3:** Univariate analyses with CCS as end-point.

Variable	Hazard ratio	(95% CI)	p-value
Age (per year)	1.04	(1.01–1.07)	0.015
Tumour size	1.41	(1.04–1.90)	0.026
FIGO stage (III-IV vs I-II)	2.54	(1.62–5.54)	0.019
Localisation (whole vs upper vagina)	3.47	(1.42–8.47)	0.010
Tumour grade (3 vs 1)	1.80	(0.66–4.89)	ns
Histology (adeno vs SSC)	0.62	(0.19–2.02)	ns
**HPV status (neg**ative **vs pos**itive**)**	4.01	(1.96–8.21)	<0.001
LRIG1 (>50% vs <50% positive cells)	0.35	(0.68–0.73)	0.011

Significant parameters were included in a Cox regression multivariate analysis, but the only factors that showed an independent, statistically significant association with patient survival were HPV status and LRIG1 expression (Table 4).

**Table 4:** Multivariate analysis Cox regression analysis.

Variable	Hazard ratio	(95% CI)	p-value
Age	1.014	(0.98–1.05)	ns
Tumour size	1.323	(0.95–5.64)	ns
FIGO stage	2.268	(0.91–5.64)	ns
HPV status	3.863	(1.81–8.25)	<0.001
LRIG1	0.408	(0.18–0.91)	0.029

## Discussion

In the present study, the expression of LRIG1, LRIG2, and LRIG3 was evaluated in a cohort of 70 PVC patients. To the best of our knowledge, this is the first study on the expression of LRIG proteins in PVC. Intriguingly, high LRIG1 expression was associated with superior cancer-specific survival in the PVC patients in our study.

LRIG proteins have previously been studied in cervical cancer, a disease with many similarities to PVC. In accordance with the present study, Lindstrom *et al* [20] showed that LRIG1 expression might be a predictor of improved survival in early-stage cervical carcinoma. In contrast to our results, where no differences between stages could be seen, LRIG1 expression appeared to decrease with increasing cervical cancer stage. Furthermore, increasing LRIG1 expression has been correlated with increasing grade of cervical intraepithelial neoplasia [32] and has also been observed as a prognostic marker in cervical adenocarcinoma [21]. Thus LRIG1 expression seems to play an important role in both precancerous and cancerous lesions.

The literature has only scarce information on the possible functions of LRIG2 and LRIG3. In 129 cases of cervical squamous cell carcinoma, LRIG2 expression was a predictor of poor prognosis in early-stage disease [27]. In addition, the combination of high LRIG2 expression and low LRIG1 expression identified women with a very poor prognosis. In contrast, no correlation was observed between LRIG2 expression and clinical parameters in cervical adenocarcinoma [21]. In the present study, no significant association was seen between LRIG2 expression and patient survival.

In an earlier study of the expression of LRIG3 in squamous cell cervical cancer, no correlation was seen with 10-year survival; however, LRIG3 expression was associated with a number of molecular events in cervical intraepithelial neoplasia [33]. It was concluded that LRIG3 was less clinically important. However, in the study by Muller *et al* [21] on cervical adenocarcinoma, having a high-fraction of LRIG3-positive cells was associated with improved patient survival. In our study, LRIG3 showed no statistically significant association with patient survival.

The role of the LRIG proteins in cancers and their association with HPV infection has been previously investigated. In our PVC samples, HPV-positive tumours showed significantly higher scores for LRIG1 than HPV-negative tumours. Lindquist *et al* showed the same positive correlation between HPV status and LRIG1 expression in oropharyngeal cancer [30]. However, we did not observe the inverse correlation between LRIG2 and HPV status that Lindquist reported. In both PVC and oropharyngeal cancer, a high expression of LRIG1 was identified and LRIG1 expression was revealed as an independent, positive prognostic factor. In addition, Lindquist *et al* demonstrated that HPV-positive tumours with high LRIG1 expression correlated with a very good prognosis in terms of disease-free survival and overall survival. This correlation between LRIG1 expression, HPV positivity, and patient survival was not significant in PVC.

In the study by Lindstrom and Hellberg, high LRIG3 expression was correlated with HPV positivity in both normal cervical epithelium and precancerous cervical lesions [33]. It was therefore suggested that LRIG3 might be involved in the development of precancerous lesions.

The most common histological type of PVC is squamous cell carcinoma, which made up the majority of our samples. In agreement with our results, previous studies on squamous cell carcinomas of the cervix and oropharynx, breast [24], skin [34], and non-small cell lung cancer [22,23] showed that high LRIG1 expression is associated with improved prognosis. In a study of squamous cell carcinoma of the skin, LRIG1 showed the highest expression in well-differentiated tumours, and these patients also proved to have the best survival [34]. In the present study, the association between LRIG expression and differentiation could not be shown, and non-keratinizing tumours were observed to have a higher LRIG1 expression, which was associated with a better prognosis. Additionally, we found that non-keratinising tumours correlated to HPV positivity. Thus our results indicate that patients with HPV-positive, non-keratinizing tumours with high LRIG1 expression might have the best prognosis.

Furthermore, it has been shown that high LRIG1 expression correlates with increased sensitivity to platinum-based chemotherapy [35], thus LRIG might be of interest as a potential predictor and target for treatment in PVC patients to minimise the risk of overtreatment.

It has been reported that LRIG ectodomains may be shed and suppress growth factor signalling in neighbouring cells, suggesting that LRIG1 ectodomains can suppress growth factor signalling in a paracrine manner [36]. This non-cell autonomous inhibition of growth factor signalling could be an interesting strategy for the treatment of PVC and other cancers. However, whether cervical and vaginal cancers depend on growth factor signalling for their growth, and whether LRIG1 can inhibit this growth, remain to be determined.

A better understanding of the role of different molecular markers in the genesis of PVC is important to optimise rational treatment interventions. Molecular markers, such as the LRIG proteins studied here, may help us to develop more efficient clinical strategies for PVC patients. Thus, analysis of LRIG expression in precursor lesions will be important to determine the potential of LRIG proteins as molecular markers for early detection and progression of invasive disease.

In summary, LRIG immunoreactivity was of prognostic importance in PVC. This provides additional knowledge about the possible clinical value of LRIG proteins in gynaecological malignancies, where LRIG1 has emerged as an independent, positive prognostic marker. LRIG proteins may be important determinants of PVC prognosis, which justifies further studies of their diagnostic and prognostic potential in this disease.

## Supporting information

S1 Table LRIG protein expression and HPV status. (DOCX)
