# Supplementary material for: Expression of LRIG proteins as possible prognostic factors in primary vaginal carcinoma
Source: PLoS One. 2017 Aug 25;12(8):e0183816. doi: 10.1371/journal.pone.0183816 (PMC5571912; doi:10.1371/journal.pone.0183816)
Supplement: S1 Table — (DOCX) [file pone.0183816.s001.docx]

**S1. LRIG protein expression and HPV status*.**

| **patient-ID** | **LRIG1 protein expression** | **LRIG2 protein expression** | **LRIG3 protein expression** | **HPV detection** | **HPV genotyping** |
| --- | --- | --- | --- | --- | --- |
| 1 | 3 | 3 | 0 | Pos | 16 |
| 2 | 3 | 3 | 0 | Neg |  |
| 3 | 3 | 3 | 0 | Neg |  |
| 4 | 3 | 3 | 1 | Pos | 16 |
| 5 | 2 | 2 | 0 | Pos | 16 |
| 6 | 3 | 3 | 1 | Neg |  |
| 7 | 2 | 2 | 0 | Neg |  |
| 8 | 3 | 3 | 0 | Pos | 16 |
| 9 | 2 | 2 | 0 | Neg |  |
| 10 | 2 | 3 | 1 | Neg |  |
| 11 | 3 | 3 | 2 | Pos | 16 |
| 12 | 3 | 3 | 0 | Insufficient material for HPV detection |  |
| 13 | 3 | 3 | 0 | Pos | 16 |
| 14 | 3 | 3 | 1 | Neg |  |
| 15 | 3 | 3 | 0 | Neg |  |
| 16 | 3 | 3 | 0 | Pos | 16 |
| 17 | 3 | 3 | 1 | Pos | 16 |
| 18 | 3 | 3 | 1 | Pos | 58 |
| 19 | 3 | 3 | 1 | Neg |  |
| 20 | 2 | 2 | 1 | Neg |  |
| 21 | 3 | 3 | 1 | Neg |  |
| 22 | 3 | 3 | 0 | Pos | 16 |
| 23 | 3 | 2 | 1 | Neg |  |
| 24 | 2 | 3 | 0 | Neg |  |
| 25 | 3 | 3 | 1 | Neg |  |
| 26 | 3 | 3 | 1 | Pos | 33 |
| 27 | 3 | 3 | 1 | Neg |  |
| 28 | 3 | 3 | 0 | Neg |  |
| 29 | 3 | 3 | 2 | Pos | 31 |
| 30 | 3 | 3 | 1 | Neg |  |
| 31 | 3 | 3 | 1 | Pos | 16 |
| 32 | 3 | 3 | 1 | Pos | 16 |
| 33 | 3 | 3 | 2 | Pos | 16 |
| 34 | 3 | 3 | 0 | Pos | 16 |
| 35 | 3 | 3 | 0 | Pos | 52 |
| 36 | 3 | 3 | 0 | Pos | 33 |
| 37 | 3 | 3 | 0 | Neg |  |
| 38 | 3 | 3 | 0 | Pos | 16 |
| 39 | 3 | 3 | 1 | Pos | 16 |
| 40 | 3 | 2 | 0 | Neg |  |
| 41 | 2 | 3 | 0 | Neg |  |
| 42 | 3 | 3 | 0 | Pos | 16 |
| 43 | 3 | 3 | 1 | Pos | 16 |
| 44 | 3 | 3 | 1 | Pos | 18 |
| 45 | 3 | 3 | 1 | Pos | 16 |
| 46 | 3 | 3 | 1 | Pos | 52 |
| 47 | 3 | 3 | 1 | Neg |  |
| 48 | 3 | 2 | 1 | Pos | 16 |
| 49 | 3 | 3 | 1 | Pos | 45 |
| 50 | 3 | 3 | 1 | Pos | 16 |
| 51 | 3 | 3 | 2 | Pos | 16 |
| 52 | 2 | 3 | 1 | Neg |  |
| 53 | 3 | 3 | 0 | Neg |  |
| 54 | 3 | 3 | 0 | Pos | 16 |
| 55 | 3 | 3 | 1 | Neg |  |
| 56 | 3 | 3 | 1 | Neg |  |
| 57 | 2 | 3 | 0 | Neg |  |
| 58 | 3 | 3 | 0 | Neg |  |
| 59 | 3 | 2 | 0 | Neg |  |
| 60 | 3 | 3 | 0 | Pos | 18 |
| 61 | 3 | 2 | 1 | Pos | 16 |
| 62 | 3 | 2 | 0 | Pos | 16 |
| 63 | 3 | 2 | 0 | Pos | 31 |
| 64 | 3 | 3 | 0 | Pos | 16 |
| 65 | 3 | 2 | 0 | Pos | 16 |
| 66 | 2 | 3 | 0 | Neg |  |
| 67 | 2 | 3 | 0 | Pos | 56 |
| 68 | 2 | 3 | 0 | Neg |  |
| 69 | 3 | 3 | 0 | Neg |  |
| 70 | 3 | 3 | 0 | Neg |  |

* **LRIG protein expression, HPV status in** 70 patients included in our study. Protein score: 0= 0%, 1= 1-25%, 2=25-50%, 3= >50% of positive cells.
